# Influence of depopulation on pollutant loads generated in the Seto inland sea basin

**DOI:** 10.1038/s41598-025-20093-8

**Published:** 2025-10-15

**Authors:** Shingo Okamoto, Yusuke Nakatani

**Affiliations:** Department of Civil Engineering, Division of Global Architecture, Graduate School of Engineering, The University of Osaka, 2-1 yamada-oka, Suita, Osaka, 565-0871 Japan

**Keywords:** Depopulation, Pollutant loads, Seto Inland Sea Basin, Phosphorus, Nitrogen, Environmental sciences, Ocean sciences

## Abstract

The Seto Inland Sea comprises both oligotrophic and eutrophic areas, requiring area-specific water management. To manage the nutrient content of the sea area, it is necessary to accurately determine the pollutant load discharged from the basin, which is believed to be significantly affected by depopulation. However, the impact of depopulation on pollutant loads is not completely clear. This study, therefore, estimated the changes in nitrogen and phosphorus pollutant loads generated in the Seto Inland Sea Basin due to depopulation from 2020 to 2050. While the population of the entire Seto Inland Sea is expected to decrease by approximately 21.1%, the amount of nitrogen is expected to decrease by approximately 12.0% and phosphorus by approximately 14.2%. The rate of decrease in the pollutant load in each sea area varied greatly, ranging from approximately 5% to 21%. The insights gained from this study will contribute to the formulation of appropriate measures for managing the water quality of the Seto Inland Sea. Moreover, they offer valuable guidance for the management of the water environment in other sea areas and their associated basins where population changes are expected.

**Supplementary Information:**

The online version contains supplementary material available at 10.1038/s41598-025-20093-8.

## Introduction

Eutrophication has been a concern in several coastal waters globally due to increased concentrations of nutrients such as nitrogen and phosphorus caused by population and industrial concentration^1–5^. Changes in nutrient concentrations significantly impact marine ecosystems, prompting several regions to adopt policies aimed at reducing the amount of pollutant loads flowing from land to sea^1,6,7^. However, eutrophication continues to progress in aquatic environments in developing countries such as India and China, where population growth and industrial concentration are advancing^8–10^. Moreover, some areas are experiencing nutrient deficiency, leading to oligotrophication in their aquatic environments^11–15^. Therefore, predicting and managing changes in pollutant load is considered crucial for implementing appropriate water environment management policies in coastal waters.

Factors that may affect pollution loads include changes in population and climate change. Numerous studies have examined the impact of climate change on the aquatic environment of lakes and sea areas, both in Japan and globally^16–21^. In contrast, research on the effects of population change has largely focused on the impact of population growth on the pollutant loads on river basins^13,22–25^. While some studies have focused on flow fluctuations and other factors related to population decline^26^, very few have explored the impact of depopulation on pollutant loads.

This study focuses on the Seto Inland Sea in Japan as a case study. The Seto Inland Sea experienced eutrophication due to the concentration of population and industry during the period of rapid economic growth from the 1950 s to the 1970s^27,28^. As a countermeasure, the Law Concerning Special Measures for Conservation of the Environment of the Seto Inland Sea was enacted in 1973, and the discharge of specified facilities into public water bodies and groundwater infiltration was regulated. Furthermore, the sewerage system was improved, and new regulatory standards were applied to domestic and industrial wastewater through the Basic Policy for Total Emission Reduction, introduced by amending the same law in 1978. In 2001, total nitrogen (T-N) and total phosphorus (T-P) were added to the regulation items, in addition to the conventional chemical oxygen demand (COD), and the number of red tide occurrences dropped from a peak of 299 in 1976 to 59 in 2022^29^. Although water quality has improved as a result of eutrophication countermeasures, there have been reports of a decrease in fish catches and fading of the color of cultivated nori in some areas of the Seto Inland Sea due to excessive reductions in nutrient concentrations, raising concerns of oligotrophication^13,30–33^. In response, the Law Concerning Special Measures for Conservation of the Environment of the Seto Inland Sea was revised in 2021 to clearly state the goal of achieving a “clean and rich sea,” promoting appropriate water environment management tailored to the actual conditions of each sea^34^.

Efforts are additionally being made to maintain appropriate nutrient levels in sea areas. In some prefectures, where environmental quality standards for water had previously set only upper limits for nutrient concentrations, new local ordinances now also stipulate lower limits. Furthermore, a nutrient management system was established in 2021, enabling the artificial supply of nutrients to the sea area^35,36^. A representative example of nutrient supply methods is the implementation of measures to increase nutrient levels at sewage treatment plants and factories. This method involves adjusting the wastewater treatment capacity or discharge treatment capacity to increase nutrient discharge concentrations compared to previous levels. It is being widely adopted in the Seto Inland Sea catchment area, starting with Hyogo Prefecture and Kagawa Prefecture, where management plans have been established.

Japan’s population began to decline in 2008, with the rate of decline accelerating since 2017. As the Seto Inland Sea has a basin that accounts for approximately 25% of the national population, the impact of depopulation cannot be ignored. It is anticipated that this will affect both pollutant loads from point and non-point sources. Nakatani et al. (2021)^37^ used a report on the calculation of pollutant loads and future population estimation data to estimate the amount of change in T-N and T-P loads from 2020 to 2050 for each bay in the Seto Inland Sea. As a result, decreases of approximately 7.0% in T-N and 9.5% in T-P were predicted for the entire Seto Inland Sea Basin. However, they focused only on the decrease in pollutant loads from domestic sources, and the impact on pollutant loads from nonpoint sources was not clear. Effective management of the aquatic environment requires controlling the amount of pollutant loads discharged into the sea, taking into account the changes due to depopulation; therefore, accurate estimation is vital. Thus, the basin presents an opportunity to evaluate how depopulation may shape future pollutant loads.

Accordingly, we evaluated the impact of depopulation on the pollutant loads. This study offers novel insights in three key aspects: (1) quantifying the impact of depopulation on pollution load, (2) considering not only the change in domestic loads but also the land use change, (3) evaluating the impact of actions to increase nutrients by comparing the increase in the amount of pollutant loads due to the active management of sewerage or factories with the decrease due to depopulation. The insights gained from this study will contribute to the formulation of appropriate measures for managing the water quality of the Seto Inland Sea. Moreover, they offer valuable guidance for managing the water environment in other sea areas and their associated basins, particularly in regions with expected population changes and declining populations in Asia and European countries^38^.

## Materials and methods

### Study area

The Seto Inland Sea is the largest enclosed sea in Japan, with a surface area of 23,203 km^2^, an average depth of 38 m, and a basin population of 29.54 million^29^. It is highly productive and has a thriving fishing industry due to its diverse topography and numerous inflowing rivers.

**Fig. 1:**
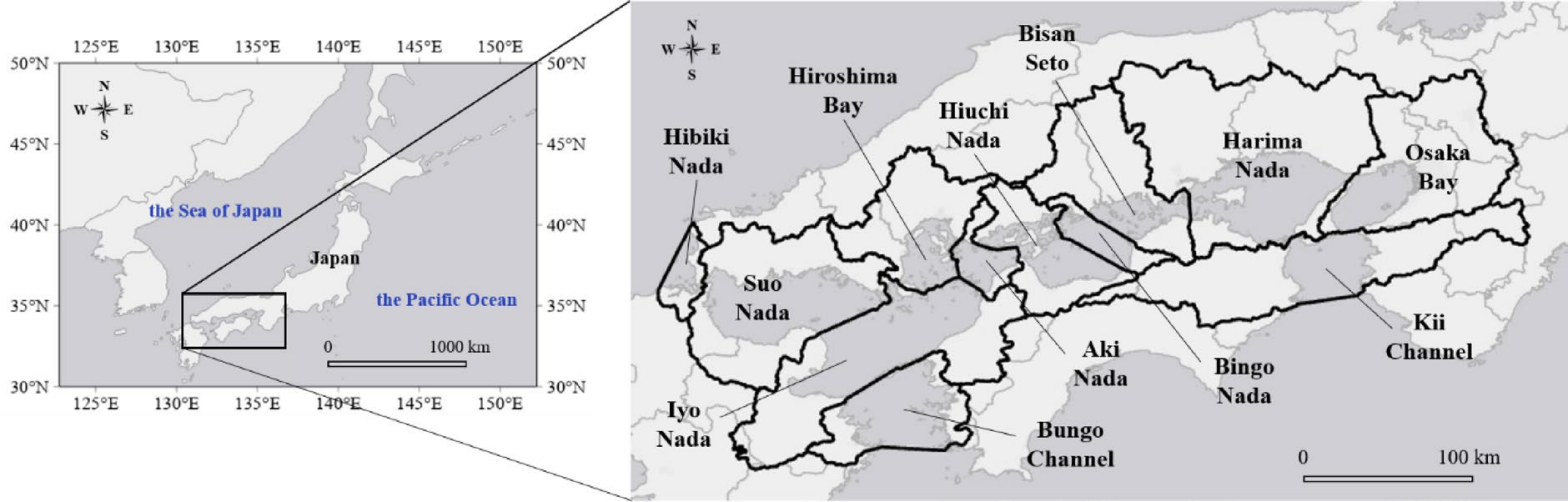
The sea boundaries of the Seto Inland Sea.

The Seto Inland Sea lies across 18 prefectures, including Shiga, Kyoto, Mie, Osaka, Hyogo, Nara, Wakayama, Tokushima, Ehime, Kagawa, Kochi, Okayama, Hiroshima, Yamaguchi, Fukuoka, Oita, Miyazaki, and Kumamoto, and environmental management is carried out by each prefecture. In addition, as presented in Fig. 1, the Seto Inland Sea comprises 12 separate bodies of water and is connected to the Pacific Ocean via the Kii and Bungo channels, and to the Sea of Japan via Hibiki Nada. This division of sea areas is based on the Law Concerning Special Measures for Conservation of the Environment of the Seto Inland Sea, and it is important to conduct water environment management that is appropriate for the actual conditions of each sea area.

In this study, the basin boundaries of each sea area were determined using GIS software (ArcGIS, ESRI) based on the law and the basin mesh data from the digital national land information^39^. Shiga, Mie, Kochi, Miyazaki, and Kumamoto prefectures are included in the river basins discharged into the Seto Inland Sea, but are not included in the basin of the Seto Inland Sea according to the division based on the Law Concerning Special Measures for Conservation of the Environment of the Seto Inland Sea.

### Data collection

We estimated the amount of pollutant loads in the fiscal year 2050 based on the reported values for T-N and T-P by prefecture in the fiscal year 2020, as listed in the report on the calculation of the amount of pollutant loads published by the Ministry of the Environment^40^. This report aims to understand the effects of aquatic pollutant load control in Tokyo Bay, Ise Bay, and the Seto Inland Sea and describes the pollutant loads of T-N and T-P by prefecture and emission source.

For the calculation of pollutant loads, population and land use data were based on the estimated future population by 1 km mesh and land use by 1 km mesh data obtained from digital national land information^41,42^. Additionally, the sewerage coverage ratio and the proportion of the population connected to the sewerage system within the treatment area were based on actual municipal data from the fiscal year 2017^43^. The industrial statistics mesh data used to calculate pollutant loads from industrial sources were from the fiscal year 2015^44^.

To assess the impact of the actions to increase nutrients, the water discharge from each sewage treatment plant and factory, and the T-N water quality of the discharge before the actions were based on the actual values in the fiscal year 2017^43^.

### Calculation of current pollutant loads

In this study, as described in Sect. Introduction, we decided to estimate the changes in pollutant loads by sea area because it is important to manage water quality according to the actual conditions of each sea area. For the current pollutant loads, the reported values by the prefecture of T-N and T-P for the fiscal year 2020, which are listed in the report, were allocated proportionally in the GIS software according to the method shown in Table 1. At this time, a 1 km mesh was used as the smallest unit. In the report, the emission sources are classified into three major categories—domestic, industrial, and others; the other category includes land, livestock, and aquaculture sources. Subsequently, we used different methods for each category. At this point, the process of discharge into the sea was not considered since the analysis in this study was focused on the pollutant loads generated in the land area.

**Table 1:** Method for allocating the amount of pollutant loads by emission source to each mesh of the calculation area.

Categories of pollutant load sources		Method
Domestic		The reported values by prefecture were allocated to each mesh in the methods shown in Table 2.
Industrial		The reported values by prefecture were allocated to each mesh based on the shipment amount by industry classification in the industrial statistics mesh data.
The other	Land (Mountains/Forests, The other lands)	The reported values by prefecture were allocated to each mesh based on the area of each land use category.
	Land (Paddies, Fields/Orchards)	Calculated by using the standard values for fertilizer application per unit area for each prefecture and the area of each land use.
	Livestock	The reported values by prefecture were allocated to each mesh based on the area of fields/orchards.
	Aquaculture	The reported values by prefecture were divided based on the number of farms in each sea area.

The pollutant loads from domestic sources for each mesh were calculated by setting the unit loads and population for each treatment type by prefecture, according to the methods presented in Table 2, where is the population connected to the sewerage system within the sewerage treatment area of a specified mesh $\:i$, is the total population in a specified mesh $\:i$, $\:{r}_{s,i}$ is the sewerage coverage ratio in a specified mesh $\:i$, $\:{r}_{c,i}$ is the rate of the population connected to the sewerage system within the sewerage treatment area of a specified mesh $\:i$, $\:{P}_{t,i,y}$ is the treatment population for each treatment type in a specified mesh $\:i$, $\:{P}_{nc,\:2020}$ is the population that did not connect to the sewerage system within the sewerage treatment area for each prefecture listed in the report, $\:{P}_{t,2020}$ is the treatment population for each treatment type and each prefecture listed in the report, $\:t$ is the treatment type, $\:y$ is the fiscal year 2020 or 2050. We considered six types of treatment—sewage treatment plants, combined septic tanks, individual septic tanks, human waste treatment plants, treated wastewater, and untreated wastewater. The percentage of the non-flushing population treated with each type of treatment was calculated using the prefectural framework described in the report.

**Table 2:** Method for setting the unit loads and treatment population for each treatment type and each mesh about pollutant loads from domestic sources.

Treatment type	Unit loads	Treatment population for each mesh
Sewage treatment plants	Calculated by dividing the reported values by the total treatment population of each prefecture.	$\:{P}_{c,i,y}$ ($\:={P}_{i,y}\cdot\:{r}_{s,i}\cdot\:{r}_{c,i}$)
Combined septic tanks	Used the unit loads by prefecture listed in the report.	$\:\left({P}_{i,y}\cdot\:{r}_{s,i}-{P}_{c,i,y}\right)\cdot\:{P}_{t,2020}/{P}_{nc,2020}$
Individual septic tanks	Used the unit loads by prefecture listed in the report.	$\:\left({P}_{i,y}\cdot\:{r}_{s,i}-{P}_{c,i,y}\right)\cdot\:{P}_{t,2020}/{P}_{nc,2020}$
Human waste treatment plants	Calculated by dividing the reported values by the total treatment population of each prefecture.	$\:\left({P}_{i,y}\cdot\:{r}_{s,i}-{P}_{c,i,y}\right)\cdot\:{P}_{t,2020}/{P}_{nc,2020}$
Treated wastewater	Used the unit loads by prefecture listed in the report.	$\:\left({P}_{i,y}\cdot\:{r}_{s,i}-{P}_{c,i,y}\right)\cdot\:{P}_{t,2020}/{P}_{nc,2020}$
Untreated wastewater	Used the unit loads by prefecture listed in the report.	$\:\left({P}_{i,y}\cdot\:{r}_{s,i}-{P}_{c,i,y}\right)\cdot\:{P}_{t,2020}/{P}_{nc,2020}$

The land load was classified into four categories—paddies, fields/orchards, mountains/forests, and other land, and the loads from paddies and fields/orchards were calculated using the unit loads in the report. At this point, in general, it is known that the pollutant loads in agricultural lands are larger from paddy fields than from fields or orchards^45–48^. However, the report applies the same unit loads of paddies and fields/orchards, and the pollutant loads from paddy fields may be overestimated. Therefore, we calculated pollutant loads in paddy fields, fields, and orchards using the fertilizer standard value per unit area in each prefecture, land use area, and fertilizer runoff rate in each mesh^49^. The fertilizer runoff rates were set based on the similarities of the crops and environments in each prefecture in the studies conducted by Takeuchi^50^. The land-use classification in the report was different from the digital national land information; therefore, we replaced it, as presented in Table 3.

**Table 3:** Land use classification compatibility.

The digital national land information	This study
Paddies	Paddies
Other agricultural lands	Fields/Orchards
Forests	Mountains/Forests
Wastelands	
Buildings	The other lands
Roads	
Railroad tracks	
Other lands	
Golf courses	

### Future scenario

To evaluate the impact of depopulation, we estimated the pollutant loads in fiscal year 2050 based on future scenarios and compared them with the value in fiscal year 2020. For the future scenario, we assumed changes in the distribution of population and land use and did not consider the effects of changes in industrial structure or climate change. Population data were obtained from existing public data, and land use was estimated based on these data. The following is a detailed description of the scenarios.

#### Scenario: population change

**Fig. 2:**
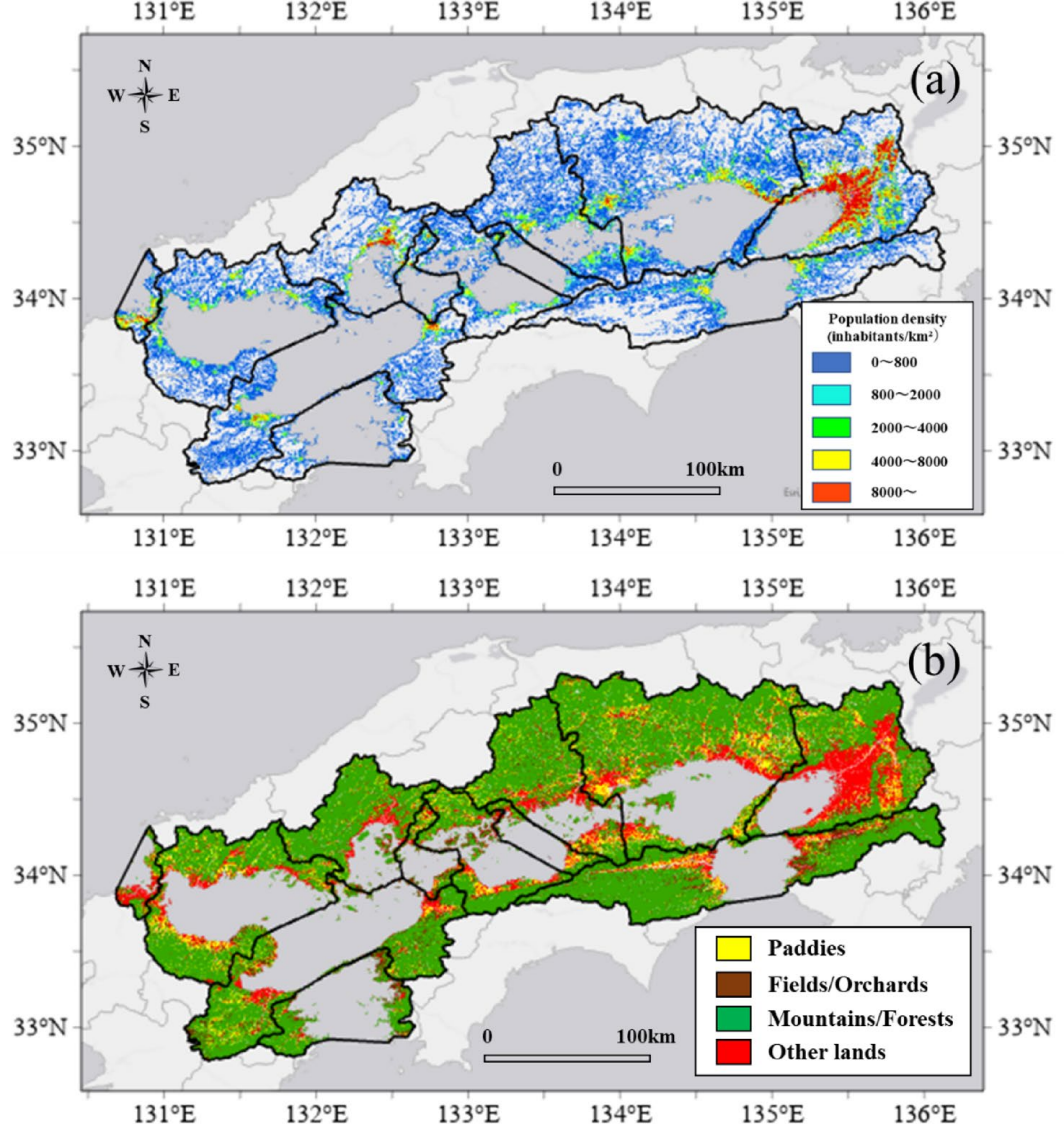
The spatial distribution of (**a**) the population in 2020, and (**b**) land use in 2020

Figure 2a presents the spatial distribution of the population in the fiscal year 2020, which is primarily concentrated in the coastal areas of Osaka Bay in the Seto Inland Sea basin. There are also large populations in the prefectural capitals of each prefecture, as well as major cities such as Shimonoseki and Kitakyushu. However, the majority of areas have a population of less than 200 people per km^2^ outside urban areas. In addition, the population is expected to increase in some urban areas and decrease in non-urban areas from 2020 to 2050, making the uneven distribution of the population more extreme.

**Fig. 3:**
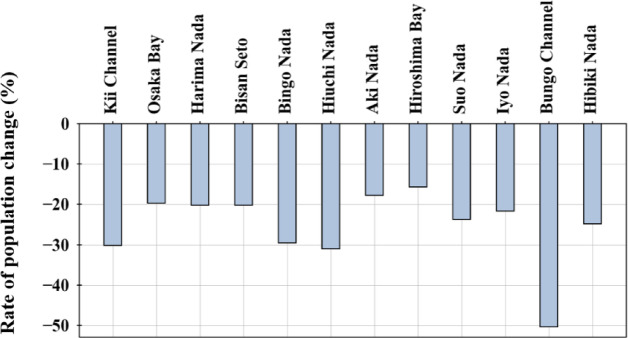
The rate of population change by sea area from 2020 to 2050

Figure 3 presents the rate of population change in the sea area from 2020 to 2050. Depopulation occurred in all sea areas, and the average decrease in the Seto Inland Sea was approximately 21.1%. The rate of change varies greatly across the sea area, generally between 15% and 30%. The population has almost halved in the Bungo Channel, in particular. The population change rate was calculated using the following formula:

1 $$\:{r}_{p,s}={(P}_{s,2050}-{P}_{s,2020})/{P}_{s,2020}$$

where, $\:{r}_{p,s}$ is the population change rate for each sea area, $\:{P}_{s,2020}$ is the population in sea area $\:s$ in 2020, and $\:{P}_{s,2050}$ is the population in sea area $\:s$ in 2050.

#### Scenario: land use change

In this study, we calculated the future land use area using the estimated future population after 2020 based on the method of Hanasaki et al.^51^ Various studies have been conducted on land-use forecasting from the perspectives of urban planning and meteorology^52–55^. The method of using transition probabilities based on past change trends, which is common in these studies, is an effective tool for land-use forecasting; however, it has the uncertainty of producing forecast results that deviate from actual phenomena in some cases. The trend of land-use area change may additionally differ when the population increases or decreases, and it is desirable to forecast land use based on data after 2008. Land use change does not occur rapidly, and it is necessary to analyze the long-term trend of land use change over several decades; however, sufficient data have not been accumulated as of 2024. It is, therefore, difficult to discuss this issue. For these reasons, we apply a method that does not use transition probabilities. The method is described as follows:

First, using the estimated future population data with a 1 km mesh, the rate of population change in each mesh was calculated from 2020 to 2050, and the area of buildings in the land use 1 km mesh data was changed in proportion to the rate of population change. When the area of buildings increased, the increase was considered a decrease in the area of mountains/forests, wastelands, paddies, and fields in the same mesh. When the area of buildings decreased, the decreased area was allocated to wastelands to keep the total area of each mesh constant. However, when the population increased and the area of buildings exceeded the total area, the area of buildings was set equal to the total area of the mesh. When the population decreased and there were no paddies, fields, mountains/forests, or wastelands in the mesh, land use was assumed to remain unchanged.

2 $$\:{r}_{p,i}={P}_{i,2050}/{P}_{i,2020}$$

3 $$\:{A}_{b,i,2050}={A}_{b,i,2020}\cdot\:{r}_{p,i}$$

where, $\:{r}_{p,i}$ is the rate of population change in mesh $\:i$ from 2020 to 2050, $\:{P}_{i,2020}$ is the total population in mesh $\:i$ in 2020, $\:{P}_{i,2050}$ is the total population in mesh $\:i$ in 2050, $\:{A}_{b,i,2020}$ is the area of building in mesh $\:i$ in 2020, $\:{A}_{b,i,2050}$ is the area of building in mesh $\:i$ in 2050. In this method, the areas of paddy fields and fields do not decrease as the population decreases. In reality, however, it was considered that agricultural land would become abandoned and eventually change to wasteland because of depopulation; thus, the future estimation of agricultural areas could not be performed correctly. Therefore, in this study, we represented the shrinkage of agricultural areas due to depopulation by decreasing the area of paddy fields in proportion to the rate of population change in each mesh.

4 $$\:{A}_{p,i,2050}={A}_{p,i,2020}\cdot\:{r}_{p,i}$$

5 $$\:{A}_{f,i,2050}={A}_{f,i,2020}\cdot\:{r}_{p,i}$$

where, $\:{A}_{p,i,2020}$ is the area of paddies in mesh $\:i$ in 2020, $\:{A}_{b,i,2050}$ is the area of paddies in mesh $\:i$ in 2050, $\:{A}_{f,i,2020}$ is the area of fields/orchards in mesh $\:i$ in 2020, $\:{A}_{f,i,2050}$ is the area of fields/orchards in mesh $\:i$ in 2050. At this point, the decrease in the area of paddy fields is not necessarily proportional to the population change in the mesh. It is considered to be closely correlated with the number of agricultural workers and the spatial characteristics of the surrounding area. Therefore, the above estimation method was considered to result in an excessive decrease in the area of city areas, paddies, and fields; however, we found no issue in presenting this as a potential future scenario because of the difficulty in accurate land use forecasts.

Figure 5b presents the spatial distribution of land use in 2021, and Table 4 compares the percentage of each land-use area in the basin by sea area in 2021 and 2050. In terms of land use classification, the classifications in the digital national land information and the report are mutually compatible, as presented in Table 3. The other land category includes several city areas; therefore, it is concentrated in urban areas and their coastal areas, and its distribution approximately corresponds to areas with large populations, as presented in Fig. 5a. In addition, paddies and fields/orchards were located around other land categories, and mountains/forests covered most areas. Looking at each sea area, the area of the other land categories was relatively large in Osaka Bay and Hibiki Nada, and the smallest proportion was in Bungo Channel. The areas of paddies and fields/orchards are relatively large in Aki Nada, Iyo Nada, and Bingo Nada, and small in Osaka Bay, Hiroshima Bay, and Hibiki Nada. Comparing 2021 and 2050, the changes in the area of paddies, fields/orchards, and other land categories are generally in line with the population change rate presented in Fig. 6; however, both paddies and fields/orchards have decreased significantly in Hiroshima Bay compared to the population change rate. This may be because the proportion of paddies and fields/orchards in the mesh where the population decreased was higher in Hiroshima Bay than in the other sea areas.

**Table 4:** A comparison of the percentage of each land use area to the basin by sea area in 2021 and 2050.

Sea area	Year	Paddies		Fields/Orchards		Mountains/Forests		The other lands	
		*A* (km^2^)	*R* (%)	*A* (km^2^)	*R* (%)	*A* (km^2^)	*R* (%)	*A* (km^2^)	*R* (%)
Kii Channel	2021	5.10	−29.35	7.01	−39.82	80.12	7.98	7.78	−27.10
	2050	3.60		4.22		86.51		5.68	
Osaka Bay	2021	8.27	−27.10	1.91	−31.19	56.18	16.03	33.64	−18.33
	2050	6.03		1.32		65.18		27.47	
Harima Nada	2021	12.41	−25.57	2.04	−30.37	72.32	8.69	13.24	−18.86
	2050	9.23		1.42		78.61		10.74	
Bisan Seto	2021	12.41	−31.01	4.51	−37.02	74.21	9.06	12.16	−18.30
	2050	9.23		2.84		80.93		9.93	
Bingo Nada	2021	11.27	−24.03	10.19	−29.98	63.21	15.66	15.34	−26.98
	2050	8.56		7.13		73.11		11.20	
Hiuchi Nada	2021	8.68	−30.36	7.36	−40.74	71.39	12.39	12.57	−25.57
	2050	6.04		4.36		80.24		9.36	
Aki Nada	2021	9.01	−17.45	14.40	−35.28	59.72	16.10	16.87	−17.55
	2050	7.44		9.32		69.33		13.91	
Hiroshima Bay	2021	4.08	−42.93	2.02	−47.93	84.08	5.45	9.82	−18.96
	2050	2.33		1.05		88.66		7.96	
Suo Nada	2021	12.16	−33.09	2.69	−33.49	73.03	9.90	12.12	−18.99
	2050	8.14		1.79		80.25		9.82	
Iyo Nada	2021	7.90	−39.13	8.57	−38.99	74.72	11.16	8.81	−21.67
	2050	4.81		5.23		83.06		6.90	
Bungo Channel	2021	2.28	−45.46	10.88	−46.78	81.22	10.52	5.62	−43.08
	2050	1.24		5.79		89.77		3.20	
Hibiki Nada	2021	6.09	−35.13	2.53	−39.53	54.89	19.22	36.50	−20.31
	2050	3.95		1.53		65.44		29.08	

Where, *A* represents the area of each land use, and *R* represents the rate of change in land use area from 2021 to 2050.

### Calculation of future pollutant loads

Based on future scenarios, we assumed that there would be an impact on domestic, land, and livestock sources. We estimated future load amounts by applying the future estimates of population and land use classification area to the method in Table 1. At this point, we assumed that the sewerage coverage rate and flushing rate would not change in the future and that the pollutant loads from industrial and aquaculture sources would not be affected by depopulation.

### Comparison with effects of actions to increase nutrients

We calculated the amount of increased loads from the amount of effluent water, the T-N quality of the effluent water before the actions, and the T-N quality of the effluent water after the actions at sewage treatment plants and factories that actively manage the actions, and compared it with the amount of decreased loads due to depopulation. Because the water quality of T-P has achieved the lower limit of nutrient levels and environmental standard values, actions to increase nutrients are primarily taken to increase T-N loads. As presented in the Online resource, the sewage treatment plants and factories considered in this study are the 37 sewage treatment plants and 8 factories in the Seto Inland Sea Basin, which are considered to be implementing actions to increase nutrients in the guidelines of Hyogo Prefecture and the Ministry of Land, Infrastructure, Transport, and Tourism^36,56^. We assumed that the amount of water released would not change and that the T-N quality of the effluent water after the actions would not exceed the quality of the water flowing into them; the value was given as the upper limit. There are several reports of cases where the increase measures are carried out seasonally in accordance with the fishing season for cultivated nori and other products in the Seto Inland Sea^56^. We, therefore, assumed that the actions at sewage treatment plants would be carried out from November to April.

## Results and discussion

### Changes in the amount of pollutant loads by sea areas

**Fig. 4:**
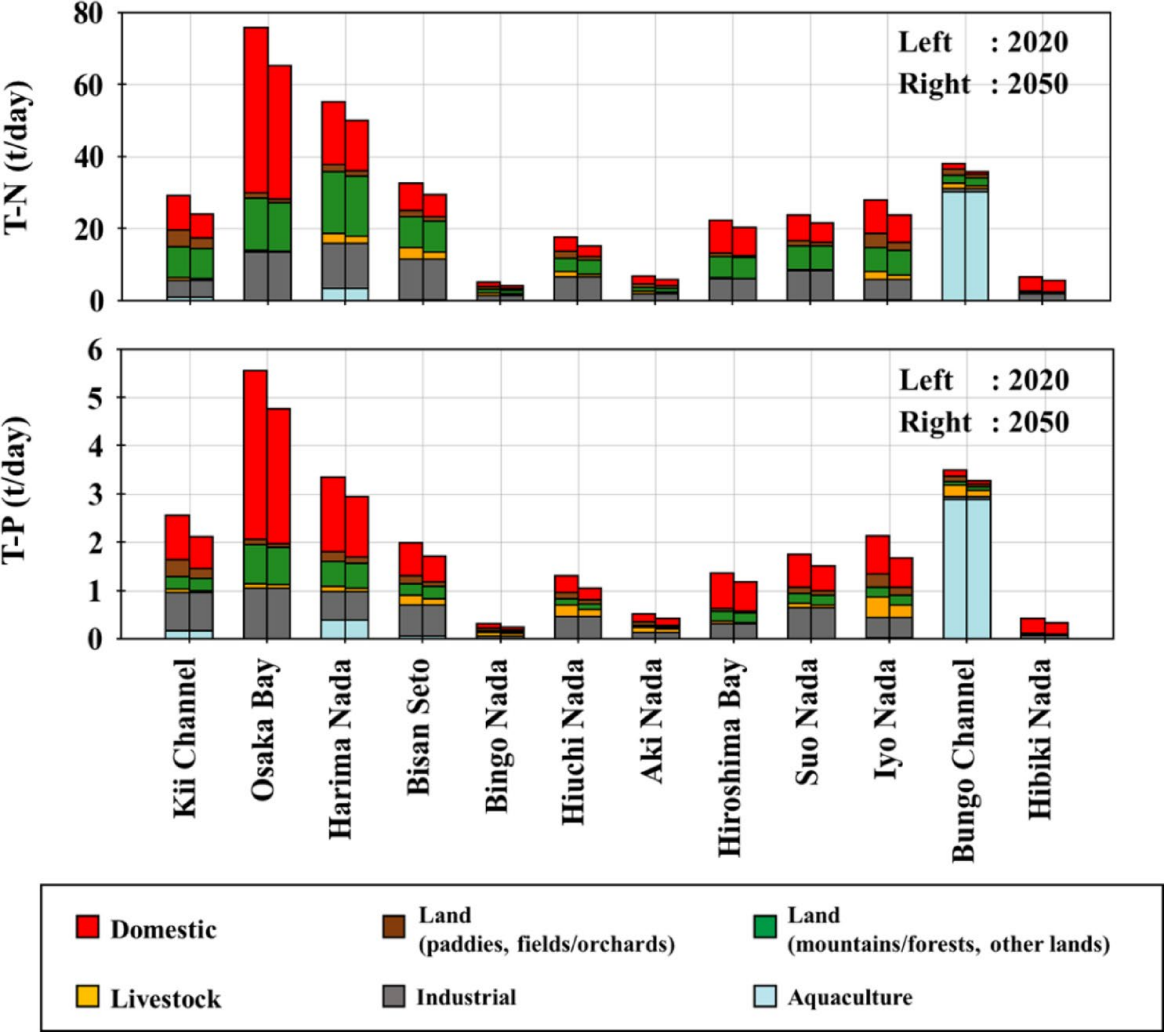
The estimated results for the T-N and T-P pollutant loads in the Seto Inland Sea from 2020 to 2050

Figure 4 presents the estimated results for the T-N and T-P pollutant loads in the Seto Inland Sea in 2020 and 2050. From 2020 to 2050, the total pollutant loads in the Seto Inland Sea decreased by approximately 40.9 tons/day (12.0%), and T-P decreased by approximately 3.52 tons/day (14.2%). Here, T-N and T-P loads are not per capita amounts, but net total values. This percentage decrease was larger in T-P than in T-N, and this was thought to be because the proportion of the total amount of pollutant loads accounted for by the pollutant loads from domestic, land, and livestock sources was higher in T-P than in T-N, which was strongly affected by depopulation and the associated land-use change. The composition of pollutant loads additionally differs greatly between sea areas. In Osaka Bay and Hibiki Nada, where there is a high concentration of people, the loads from domestic sources accounted for approximately 60.6% of the pollutant loads, whereas in Bungo Channel, the loads from aquaculture sources accounted for approximately 79.7%, indicating the geographical and cultural characteristics of each sea area.

**Fig. 5:**
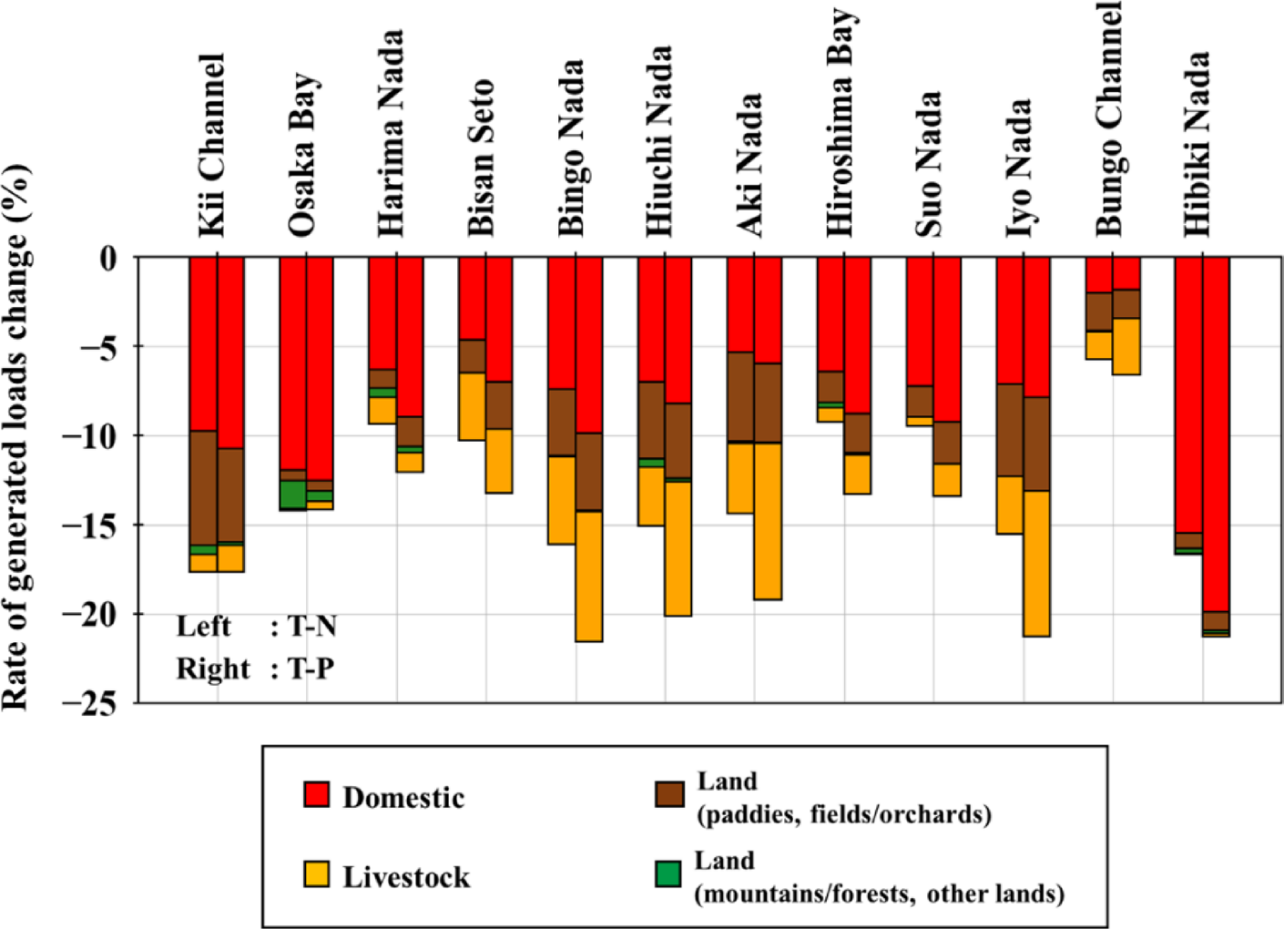
The rate of change in the amount of pollutant loads by sea areas from 2020 to 2050

Figure 5 presents the rate of change in pollutant load by sea area from 2020 to 2050. In Osaka Bay and Hibiki Nada, even though the population and the area classified as other land is large, the depopulation rate is relatively small. The majority of the change in the amount of pollutant loads is due to changes in the amount of load from domestic sources; in summary, the amount of loads is expected to decrease significantly. In the Bungo Channel, where the majority of the load came from aquaculture sources, the population decreased by approximately 50.3%; however, the decrease in load was the smallest. In the Kii Channel, Bingo Nada, Hiuchi Nada, Aki Nada, and Iyo Nada, the loads from land sources account for more than 10% of the total loads. In particular, the land use areas of paddies, fields, and orchards in Bingo Nada, Aki Nada, and Iyo Nada are relatively large, and the rate of load decrease is also high. Therefore, it was thought that the loads from land sources (paddies and fields/orchards) decreased significantly compared to other sea areas because of the significant impact of land use change. For the same reason, livestock sources have also been affected by land-use changes and have decreased significantly.

The decrease in the amount of pollutant loads estimated by Nakatani et al.^19^ was based on calculations that only took into consideration changes in the loads from domestic sources. However, in this study, in addition to this, changes in the loads from land and livestock sources were also taken into consideration, and a decrease of approximately 15.02 tons per day in T-N (4.40%) and approximately 1.35 tons per day in T-P (5.44%) was expected.

In terms of the amount of loads discharged into the Seto Inland Sea, Nakada et al.^57^ calculated the changes of pollutant loads due to climate change based on the RCP8.5 scenario, considering the change of precipitation, and using a distributed runoff model. They reported that T-N loads are expected to increase by approximately 0.80% (2.52 t/day) and T-P loads by approximately 4.13% (1.09 t/day) from the end of the 20th century to the end of the 21 st century. This prediction is the result of an estimate of the amount of runoff into the sea, and the time frame is different, so it is not possible to directly compare it with the results of this study. However, the decrease in the amount of pollutant loads estimated in this study greatly exceeds the increase due to climate change. It was found that the impact of the decrease in population and accompanying changes in land use cannot be ignored.

### Correlation between rate of decrease in pollutant loads and rate of depopulation

**Fig. 6:**
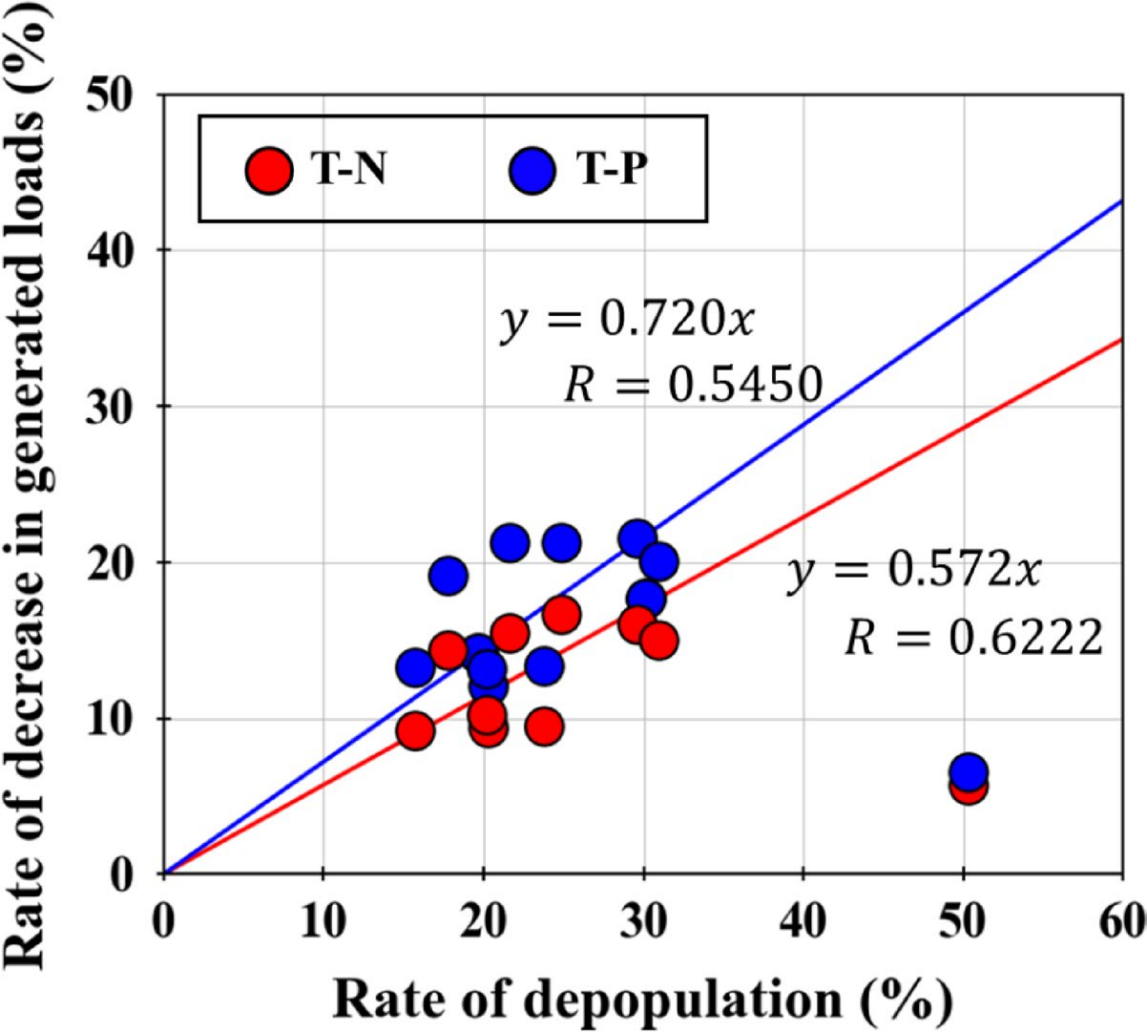
The relation between the rate of decrease in the amount of generated loads and the rate of depopulation by sea area from 2020 to 2050

Figure 6 presents the relationship between the rate of decrease in pollutant loads and the rate of depopulation by sea area from 2020 to 2050. There was no clear correlation between the rate of decrease in loads and the rate of depopulation when all 12 sea areas in the Seto Inland Sea basin area were considered. However, there was a positive correlation when 11 sea areas (excluding the Bungo Channel) were considered. This is because loads from aquaculture sources account for most of the total pollutant loads in the Bungo Channel and are not easily affected by depopulation. We obtained the relationship equations presented in Fig. 6 for T-N and T-P, and there were sea areas where the rate of decrease in pollutant loads was estimated to be relatively high or low around this relationship equation. This trend is generally explained by the proportion of loads from livestock, land (paddies, fields/orchards), and domestic sources to the total pollutant loads for each sea area.

### Comparison with the actions to increase nutrients

**Fig. 7:**
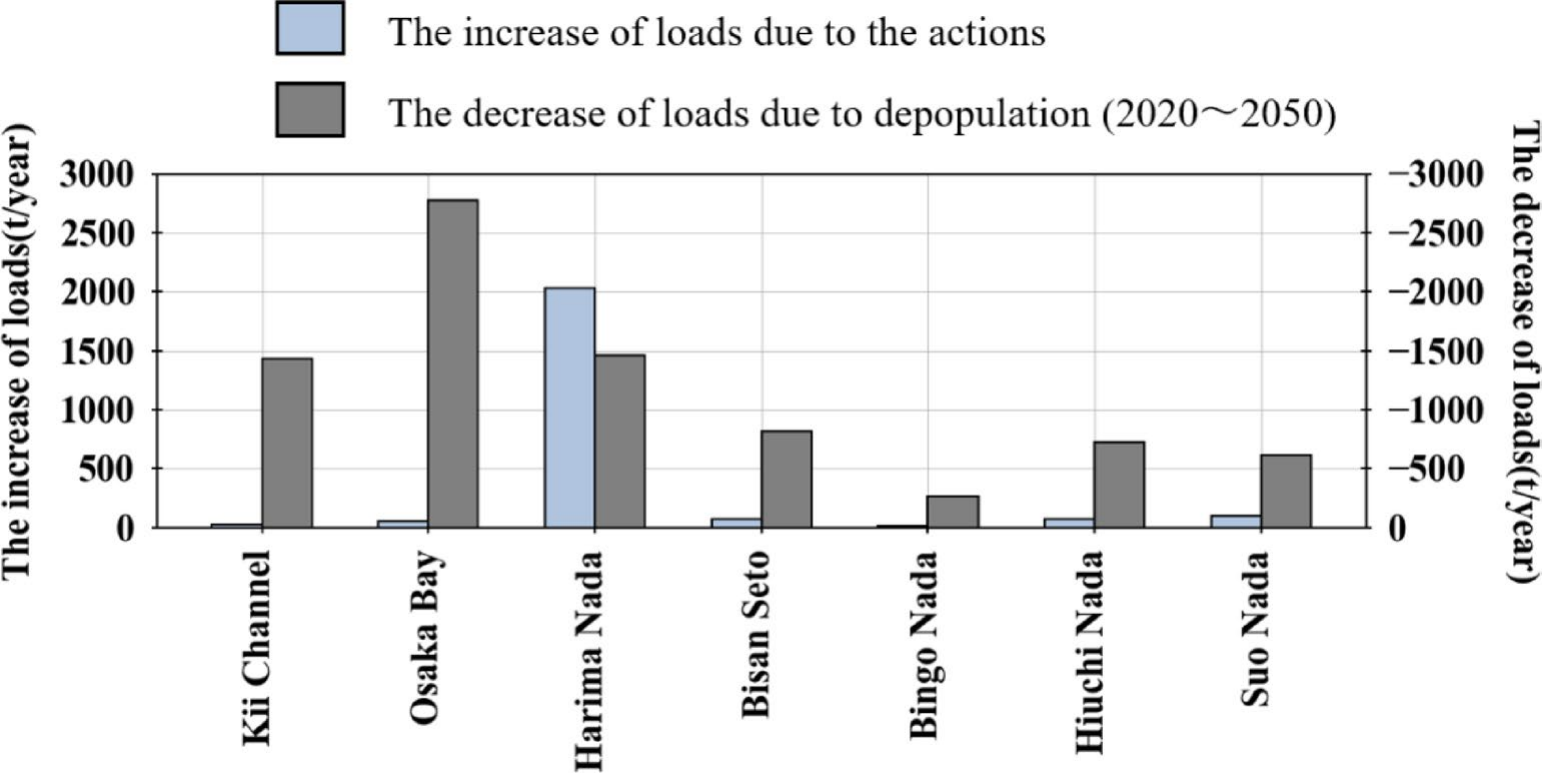
The comparison of the increase in loads due to the actions to increase nutrients and the decrease in loads due to depopulation

Figure 7 presents a comparison of the increase in loads due to the actions taken to increase nutrients and the decrease in loads due to depopulation. The results of the calculation of the increase in loads due to the actions to increase nutrients showed that the inflow loads to the sea area in Harima Nada increased by approximately 2,029 tons/year in the year after the actions compared to the year before the actions were taken in Harima Nada. Based on Sect. Changes in the amount of pollutant loads by sea areas, the pollutant loads in Harima Nada will decrease by approximately 1890 t/year from 2020 to 2050, and by approximately 1,459 t/year in the sea area, using the delivery rate to the sea area in the report. Therefore, in Harima Nada, the increase in loads due to the actions outpaced the decrease in loads due to depopulation, and it was found that there was an impact from current actions. However, if the population continues to decline after 2050, the decrease in load due to depopulation will likely exceed the increase in load owing to these actions. This study used the inflow and planned water quality as the effluent water quality after the actions, but actual cases have been reported to have water quality values that are lower than these values^56^. Therefore, the increase in load may be an overestimation. The decrease and increase in loads are certainly at least of the same order of values.

In sea areas other than Harima Nada, the increase was approximately 54 t/year in Osaka Bay, 29 t/year in Kii Channel, 67 t/year in Bisan Seto, 21 t/year in Bingo Nada, 72 t/year in Hiuchi Nada, and 99 t/year in Suo Nada. In these areas, even after considering the delivery rate, the values were significantly smaller than the decrease in load due to depopulation by 2050. This may be due to the following three reasons: (1) there are fewer sewage treatment plants and factories than in Harima Nada; (2) the volume of treated water is smaller because of the smaller population; and (3) there are fewer sewage treatment plants and factories implementing actions to increase nutrients. Therefore, the results suggest the need for appropriate nutrient management, other than that at Harima Nada, as part of future water environment management measures.

A non-negligible decrease in the loads was expected compared to the increase in the loads due to climate change and actions to increase nutrients. This suggests that pollutant loads may change when the population changes not only in the Seto Inland Sea basin but also in other basins, and it is important to propose measures that take such effects into account. Therefore, it is important to investigate the rate of increase or decrease in population and the composition of population loads, focusing on the sufficiency of nutrient concentrations in the sea area.

### Limitations of the analysis and assessment

This study estimated changes in pollutant load quantities associated with population changes from 2020 to 2050. The estimates were calculated based on several assumptions; errors corresponding to the assumptions used may, therefore, occur.

Estimates of pollutant load quantities and land use changes were based on projected future population figures. The estimated population is based on values published by the digital national land information at 1 km mesh intervals. However, errors may arise from the use of the cohort component method, as well as from unforeseeable external factors such as disasters or pandemics. Kamata et al.^58^ reported that when using the cohort component method to predict the population in 2015 based on 1985 as the base year, an error of approximately 8.5% occurred when predicting the population by prefecture. This is attributed to Japan’s declining population and the decreasing trend in the population of younger age groups, which have a higher error rate. The population data used in this study was estimated based on the 2015 survey results and is expected to include errors of approximately this magnitude. Changes in pollutant load due to population changes may also have a similar or less range of variability.

In terms of future sewerage coverage rates and flush toilet rates, the estimates for 2050 assume no change from current levels. The national average sewerage coverage rate has remained at approximately 80% since 2017, while the flush toilet rate has remained at approximately 92%. Although slight annual increases are observed for both rates, these are considered negligible for this study’s future projections.

In terms of changes in land use, this estimate is based on the assumption that farmland decreases in proportion to population, as mentioned in 2.4.2. However, in reality, various factors such as economic shifts, aging, and surrounding spatial characteristics are thought to have an influence. In addition, although data for 2017 are provided for the sewerage coverage rate and flushing rate, these values are also expected to change in the future. Therefore, in order to make more accurate estimates, it is necessary to take these effects into consideration. In comparison with the actions to increase nutrients, it should be noted that the effectiveness of actions to increase nutrients should be evaluated considering the flow and water quality after inflow into the sea area.

Although we estimated the change in pollutant loads focusing on the effects of depopulation, it is important to include changes in industrial and aquaculture loads in the calculations, considering factors such as industrial structure and climate change for a precise discussion of water management measures in the sea area. It is also vital to evaluate the effects of changes in pollutant loads on the flow and water quality in sea areas.

## Conclusions

In this study, we estimated the change in pollutant loads from 2020 to 2050 due to depopulation in the Seto Inland Sea Basin. As a result, in the Seto Inland Sea as a whole, it was found that loads would decrease by approximately 40.9 t/day in T-N and approximately 3.52 t/day in T-P, which are approximately 12.0% and 14.2% less than those in 2020, respectively, taking into account changes in population and land use distribution due to depopulation. The decrease in the pollutant loads was found to be a combined effect of approximately 7.60% and 8.76% decreases due to changes in the loads from domestic sources and approximately 4.40% and 5.44% decreases due to changes in land use for T-N and T-P, respectively. The rate of change in the pollutant loads generally varied from 5% to 21% for each sea area, along with the composition of the sources. Comparing the rate of population and decrease in loads in each sea area, a positive correlation was observed in 11 sea areas (excluding Bungo Channel), indicating that a 10% decrease in depopulation can result in a 5.7% decrease in T-N and a 7.2% decrease in T-P. This indicates that phosphorus is more sensitive to depopulation than nitrogen. Comparing the decrease in loads due to depopulation and the increase in loads due to actions to increase nutrients, the decrease in loads was approximately the same order of magnitude in Harima Nada, In contrast, the decrease in loads was much greater than the increase in loads in the other sea areas.

The necessary matters for the management of pollution loads and water quality are specified in the Ninth Basic Policy for Total Emission Reduction in 2022^59^. The basic policy states that the detailed management of each sea area and season is necessary based on the actual use of the sea area in the region. The policy also states that the development of sewage facilities, the promotion of measures to improve bottom sediment quality in deep bays, and the establishment of aquaculture with less environmental impact are among the matters related to pollution loads; however, it does not clearly state the impact of depopulation. However, this study shows that the impact of depopulation on pollutant loads in the Seto Inland Sea is significant, and the proportion varies from sea to sea. Therefore, the impact of depopulation should be considered when discussing future aquatic environment management measures.

## Supplementary Information

Below is the link to the electronic supplementary material.

Supplementary Material 1

## Data Availability

The pollution load data used in this study were provided by the Ministry of the Environment of Japan in electronic format upon request. This data are also published in print and can be accessed by the public through the National Diet Library and other institutions, as cited in the references. Other data—including 1 km-mesh population and land use data, municipal sewerage coverage and connection rates, industrial statistics mesh data, and effluent discharge volumes and T-N concentrations—are publicly available and can be accessed by anyone. Details are provided in the Methods section and corresponding references.

## References

[1] Boesch, D. F. Challenges and opportunities for science in reducing nutrient over-enrichment of coastal ecosystems. *Estuaries* **25**, 886–900. 10.1007/BF02804914 (2002).

[2] Gorgoglione, A. et al. Influence of land use/land cover on surface-water quality of Santa Lucia river, Uruguay. *Sustainability* **12**, 4692. 10.3390/su12114692 (2020).

[3] Moal, M. L. et al. Eutrophication: A new wine in an old bottle? *Sci. Total Environ.* **651** (15), 1–11. 10.1016/j.scitotenv.2018.09.139 (2019). 30223216 10.1016/j.scitotenv.2018.09.139

[4] Nixon, S. W. Coastal marine eutrophication: A definition, social causes, and future concerns. *Ophelia* **41**, 199–219. 10.1080/00785236.1995.10422044 (1995).

[5] Seitzinger, S. P. et al. Global river nutrient export: A scenario analysis of past and future trends. *Global Biogeochem. Cycles* **24**, GB0A08. 10.1029/2009GB003587 (2010).

[6] Cozzi, S., Ibáñez, C., Lazar, L., Raimbault, P. & Giani, M. Flow regime and nutrient-loading trends from the largest South European watersheds: implications for the productivity of mediterranean and black sea’s coastal areas. *Water* **11**. 10.3390/w11010001 (2019).

[7] Duarte, C. M., Conley, D. J. & Carstensen, J. Sánchez-Camacho, M. Return to *Neverland*: shifting baselines affect eutrophication restoration targets. *Estuaries Coasts*. **32**, 29–36. 10.1007/s12237-008-9111-2 (2009).

[8] Qin, B., Kong, X., Wang, R., Zhao, Y. & Yang, X. Lake restoration time of lake Taibai (China): A case study based on paleolimnology and ecosystem modeling. *J. Paleolimnol*. **68**, 25–38. 10.1007/s10933-020-00165-7 (2022).

[9] Ramesh, R., Robin, R. S. & Purvaja, R. An inventory on the phosphorus flux of major Indian rivers. *Curr. Sci.* **108**, 1294–1299. http://www.jstor.org/stable/24905491 (2015).

[10] Sarma, V. V. S. S. et al. Intra-annual variability in nutrients in the Godavari estuary, India. *Contin Shelf Res.* **30**, 2005–2014. 10.1016/j.csr.2010.10.001 (2010).

[11] Ibáñez, C. et al. Ecosystem-level effects of re-oligotrophication and N:P imbalances in rivers and estuaries on a global scale. *Glob. Change Biol.* **29**, 1248–1266. 10.1111/gcb.16520 (2023). 10.1111/gcb.16520 PMC10107953 36366939

[12] Derolez, V. et al. Two decades of oligotrophication: evidence for a phytoplankton community shift in the coastal lagoon of thau (Mediterranean sea, France). *Estuar. Coastal. Shelf Sci.* **241**, 106810. 10.1016/j.ecss.2020.106810 (2020).

[13] Fujiwara, T. Cultural oligotrophication in enclosed coastal seas -Responses of primary production and nitrogen dynamics to nutrient reduction-. *Bull. Coast Oceanogr.* **52**, 11–27. 10.32142/engankaiyo.52.1_11 (2014).

[14] Bernát, G. et al. Oligotrophication of lake Balaton over a 20-year period and its implications for the relationship between phytoplankton and zooplankton biomass. *Hydrobiologia* **847**, 3999–4013. 10.1007/s10750-020-04384-x (2020).

[15] Evans, M. A., Fahnenstiel, G. & Scavia, D. Incidental oligotrophication of North American great lakes. *Environ. Sci. Technol.* **45**, 3297–3303. 10.1021/es103892w (2011). 21417221 10.1021/es103892w

[16] Hao, L. et al. Long-term developments in seasonal hypoxia and response to climate change: A three-decade modeling study in the Ariake sea, Japan. *Sci. Total Environ.* **929**, 172471. 10.1016/j.scitotenv.2024.172471 (2024). 38615772 10.1016/j.scitotenv.2024.172471

[17] Higashi, H., Yoshinari, H., Nakada, S., Yokoyama, A. & Koshikawa, H. Climate change impacts on nitrogen flow in Harima Nada under RCP8.5 scenarios. *J.J. JSCE Ser. B1.* **77**, I_1099–I_1104. 10.2208/jscejhe.77.2_I_1099 (2021).

[18] Maruya, Y. et al. Uncertainty of regional climate model and impact assessment model toward climate change impact assessment. *J.J. JSCE Ser. B1.* **74**, I_109–I_114. 10.2208/jscejhe.74.5_I_109 (2018).

[19] Adrian, R. et al. Monica W. Lakes as sentinels of climate change. *Limnol. Oceanogr.* **54**, 2283–2297. 10.4319/lo.2009.54.6_part_2.2283 (2009). 20396409 10.4319/lo.2009.54.6_part_2.2283 PMC2854826

[20] Shimizu, Y. & Onodera, S. Effects of climate change on nutrient discharges in suburban watershed. *Jpn J. Limnol.* **73**, 235–254. 10.3739/rikusui.73.235 (2012).

[21] Whitehead, P. G., Wilby, R. L., Battarbee, R. W., Kernan, M. & Wade, A. J. A review of the potential impacts of climate change on surface water quality. *Hydrol. Sci. J.* **54**, 101–123. 10.1623/hysj.54.1.101 (2009).

[22] Kimbi, S. B., Onodera, S., Wang, K., Kaihotsu, I. & Shimizu, Y. Assessing the impact of urbanization and climate change on hydrological processes in a suburban catchment. *Environments* **11**, 225. 10.3390/environments11100225 (2024).

[23] Mello, K. et al. Multiscale land use impacts on water quality: assessment, planning, and future perspectives in Brazil. *J. Environ. Manage.* **270**, 110879. 10.1016/j.jenvman.2020.110879 (2020). 32721318 10.1016/j.jenvman.2020.110879

[24] Qian, Y. et al. The response of the migration of non-point source pollution to land use change in a typical small watershed in a semi-urbanized area. *Sci. Total Environ.* **785**, 147387. 10.1016/j.scitotenv.2021.147387 (2021).

[25] Yanagihara, H. et al. Nationwide evaluation of changes in fluvial and pluvial flood damage and the effectiveness of adaptation measures in Japan under population decline. *Int. J. Disaster Risk Reduc.* **110**, 104605. 10.1016/j.ijdrr.2024.104605 (2024).

[26] Chiba, K. & Kazama, S. Assessment of changes in the flow of small river basins due to depopulation using a distributed runoff model. *J.J. JSCE Ser. B1.* **78**, I_535–I_540. 10.2208/jscejhe.78.2_I_535 (2022).

[27] Imai, I., Yamaguchi, M. & Hori, Y. Eutrophication and occurrences of harmful algal blooms in the Seto inland sea, Japan. *Plankton Benthos Res.* **1**, 71–84. 10.3800/pbr.1.71 (2006).

[28] International EMECS Center. Environmental Guidebook on the Enclosed Coastal Seas of the World, 53–59. https://www.emecs.or.jp/en/wp-content/uploads/2019/10/2015_guidebook-10_setoinlandsea.pdf (2015).

[29] Fisheries Agency & Fisheries Management Office. Red tide in the Seto Inland Sea. https://www.jfa.maff.go.jp/setouti/akasio/index.html (2023).

[30] Fujiwara, T., Kobayashi, S., Kunii, M. & Uno, N. Nitrogen and phosphorus in Seto inland sea: their origin, budget and variability. *Bull. Coast Oceanogr.* **43**, 129–136. 10.32142/engankaiyo.43.2_129 (2006).

[31] Yamamoto, T. Oligotrophication in the Western Seto inland sea. *Bull. Coast Oceanogr.* **52**, 49–58. 10.32142/engankaiyo.52.1_49 (2014).

[32] Yamamoto, T. The Seto inland Sea – Eutrophic or oligotrophic? *Mar. Pollut Bull.* **47**, 37–42. 10.1016/S0025-326X(02)00416-2 (2003). 12787595 10.1016/S0025-326X(02)00416-2

[33] Yanagi, T. Management of nutrient concentration in coastal seas. *Bull. Coast Oceanogr.* **58**, 11–18. 10.32142/engankaiyo.2020.8.001 (2020).

[34] Ministry of the Environment. Regarding the law to amend law concerning special measures for conservation of the environment of the Seto inland sea. https://www.env.go.jp/water/heisa/setonaikai_law_rev.html (2022).

[35] Ministry of Land, Infrastructure, Transport and Tourism. The guidelines for the effective implementation of active operation and management of nutrients. https://www.mlit.go.jp/mizukokudo/sewerage/content/001597388.pdf (2023).

[36] Ministry of Land, Infrastructure, Transport and & Tourism The report of study group on the future of sewerage management in an era of maintenance and management under conditions of Depopulation. https://www.mlit.go.jp/mizukokudo/sewerage/content/001371608.pdf (2020).

[37] Nakatani, Y., Ono, K., Kashima, C. & Nishida, S. Estimation of pollutant loads of nitrogen and phosphorus in the Seto inland sea considering the change of domestic loads due to depopulation. *J. JSCE Ser. G*10.2208/jscejer.77.6_II_67 (2021) (**II_67–II_72**).

[38] United Nations Department of Economic and Social Affairs Population Division. World Population Prospects. Standard Projections, Most Used, Compact File, Estimates tab, Population Growth Rate (percentage) column (2024). https://population.un.org/wpp/downloads?folder=Standard%20Projections&group=Most%20used

[39] Ministry of Land, Infrastructure, Transport and Tourism. The digital national land information, the basin mesh data. (2009). https://nlftp.mlit.go.jp/ksj/gml/datalist/KsjTmplt-W07.html

[40] Ministry of the Environment. The Report on the Calculation of the Amount of Pollutant Loads Regarding Aquatic Pollutant Load Control (2022).

[41] Ministry of Land, Infrastructure, Transport and Tourism. The digital national land information, the estimated future population by 1 km mesh. https://nlftp.mlit.go.jp/ksj/gml/datalist/KsjTmplt-mesh1000h30.html (2018).

[42] Ministry of Land, Infrastructure, Transport and Tourism. The digital national land information, the land use data by 1 km mesh. https://nlftp.mlit.go.jp/ksj/gml/datalist/KsjTmplt-L03-a-2021.html (2021).

[43] Japan Sewage Works Association. Sewerage Stat. Data Fisc 2017. **74** (2019).

[44] Research Institute of Economy, Trade and Industry. The Industrial Statistics Mesh Data Fiscal. 2010. (2015)

[45] Haruta, S. et al. Effluent N, P and COD loads from paddy fields in japan: A critical review. *Mizu Kankyo Gakkaishi*. **38**, 81–91. 10.2965/jswe.38.81 (2015).

[46] Kawashima, H. et al. Estimation of pollution load generated from paddy fields. *Jpn Agric. Syst.* **14**, 1–5. 10.14962/jass.14.1_1 (1998).

[47] Kunimatsu, T. & Takeda, I. Estimation of nutrient loading from agricultural lands and forest. *Japan J. Water Pollution Res.* **11**, 743–747. 10.2965/jswe1978.11.743 (1988).

[48] Takeuchi, M. Nitrogen budgets in agricultural fields in rela-tion to their characteristics in the water purification. *Bull. Jpn Soc. Fish. Oceanogr.* **61**, 187–189 (1997).

[49] Ministry of Agriculture, Forestry and Fisheries. Fertilizer application standards for each prefecture. https://www.maff.go.jp/j/seisan/kankyo/hozen_type/h_sehi_kizyun/index.html (2020).

[50] Takeuchi, M. Nitrate and phosphate outflow from arable land. *Soil. Sci. Plant. Nutr.* **68**, 708–715. 10.20710/dojo.68.6_708 (1997).

[51] Hanasaki, N., Takahashi, K. & Hijioka, Y. Climate and socioeconomic scenarios for climate change impact and adaption assessments in Japan. *Environ. Sci.* **25**, 223–236. 10.11353/sesj.25.223 (2012).

[52] Dadashpoor, H., Azizi, P. & Moghadasi, M. Land use change, urbanization, and change in landscape pattern in a metropolitan area. *Sci. Total Environ.* **655**, 707–719. 10.1016/j.scitotenv.2018.11.267 (2019). 30476851 10.1016/j.scitotenv.2018.11.267

[53] Lambin, E. F., Geist, H. J. & Lepers, E. Dynamics of land-use and land-cover change in tropical regions. *Annu. Rev. Environ. Resour.* **28**, 205–241. 10.1146/annurev.energy.28.050302.105459 (2003).

[54] Kalnay, E. & Cai, M. Impact of urbanization and land-use change on climate. *Nature* **423**, 528–531. 10.1038/nature01675 (2003). 12774119 10.1038/nature01675

[55] Turner, B., Meyer, W. B. & Skole, D. L. Global land-use/land-cover change: towards an integrated study. *Ambio* **23**, 91–95. http://www.jstor.org/stable/4314168 (1994).

[56] Environment Department & Water and Air Division. Hyōgo Prefecture. The guidelines (knowledge base) for the supply of nutrients at factories and workplaces. https://www.kankyo.pref.hyogo.lg.jp/application/files/9117/1627/7973/0d254c92abf0a5906b9f7e7c739e3e24.pdf (2024).

[57] Nakada, S., Higashi, H., Akiyama, C. & Yoshinari, H. Climate change impact on pollutant loads from Seto inland sea basin using RCP8.5 scenarios. *J. J. JSCE Ser. B1*. **76**, I_1429–I_1434. 10.2208/jscejhe.76.2_I_1429 (2020).

[58] Kamata, K., Koike, S. & Yamauchi, M. A study on the evaluation the accuracy of future population estimated by region -Verification of spatial characteristics of estimation errors-. *Research Report on Population Analysis, Future Projections, and Applications in Response to New Trends of Declining Birthrates and Aging: International and Regional Perspectives*, 195–214. https://mhlw-grants.niph.go.jp/system/files/2017/171011/201701011A_upload/201701011A0020.pdf (2018).

[59] Ministry of the Environment. Basic policy on reduction of chemical oxygen demand, nitrogen content, and phosphorus content (Seto inland Sea). https://www.env.go.jp/content/900518423.pdf (2022).

