# Supplementary material for: Influence of depopulation on pollutant loads generated in the Seto inland sea basin
Source: Sci Rep. 2025 Oct 15;15:36078. doi: 10.1038/s41598-025-20093-8 (PMC12528400; doi:10.1038/s41598-025-20093-8)
Supplement: Supplementary file 1 — Supplementary Material 1 [file 41598_2025_20093_MOESM1_ESM.pdf]

Influence of Depopulation on Pollutant Loads Generated in The Seto Inland Sea Basin

Shingo Okamoto, Yusuke Nakatani*

Department of Civil Engineering, Division of Global Architecture, Graduate School of Engineering, The University of Osaka, Japan

* Corresponding author

E-mail: nakatani@civil.eng.osaka-u.ac.jp

Supplementary Materials

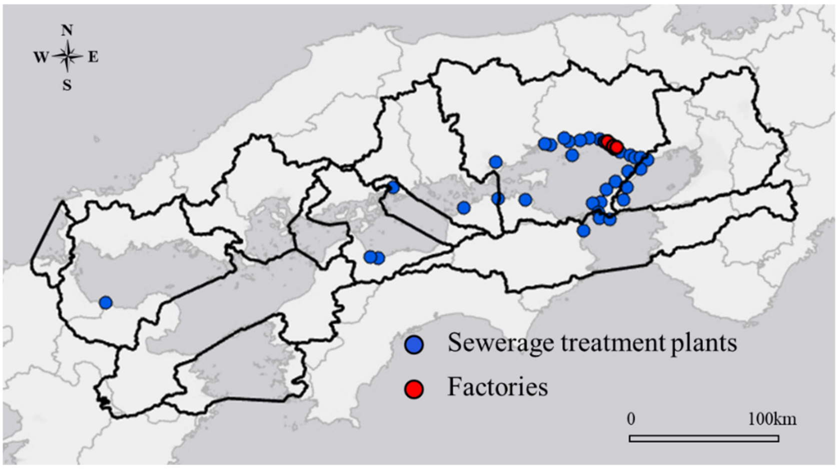

Figure S1 The sewage treatment plants and factories considered in this study implementing the actions to increase nutrients.

Table S1 The amount of fertilizer in each prefecture.

Prefecture	T-N (kg/10a)	T-P (kg/10a)
Kyoto	32.2	16.4
Osaka	23.7	16.5
Hyogo	20.1	15.8
Nara	25.1	15.9
Wakayama	21.5	14.9
Okayama	16.4	12.1
Hiroshima	23.1	16.1
Yamaguchi	20.4	14.7
Tokushima	23.1	17.3
Kagawa	21.9	15.0
Ehime	21.9	15.3
Fukuoka	16.1	11.2
Oita	20.9	15.1

Table S2 The reported T-N pollutant loads (kg/day) by prefecture used in the method described in Table 1.

Prefecture	Domestic	Industrial	Land (Mountains/Forests, The other lands)	Land (Paddies, Fields/Orchards)	Livestock	Aquaculture
Kyoto	684	318	84	8	6	0
Osaka	1593	356	87	13	90	9
Hyogo	1419	544	275	66	7	121
Nara	439	171	80	17	6	5
Wakayama	420	408	73	19	3	61
Okayama	842	461	322	64	150	0
Hiroshima	1085	496	276	31	289	33
Yamaguchi	677	686	206	34	16	10
Tokushima	494	385	174	16	71	153
Kagawa	516	358	76	32	144	345
Ehime	661	483	191	66	661	2173
Fukuoka	281	130	46	12	4	0
Oita	595	478	212	50	330	588

Table S2 The reported T-N pollutant loads (kg/day) by prefecture used in the method described in Table 1.

**Categories of sources**	**Kyo.**	**Osa.**	**Hyo.**	**Nar.**	**Wak.**	**Oka.**	**Hir.**	**Yam.**	**Tok.**	**Kag.**	**Ehi.**	**Fuk.**	**Oit.**
Domestic(1)	5681	22064	16063	1992	375	1984	8472	3062	316	972	2099	3142	2189
Domestic(2)	67	201	463	259	490	356	326	128	351	100	170	197	156
Domestic(3)	8	8	14	23	14	33	3	6	63	0	18	0	3
Domestic(4)	12	21	5	2	11	26	18	11	19	3	6	4	6
Domestic(5)	0	924	0	684	0	0	114	138	2	58	0	0	1956
Domestic(6)	136	0	372	0	814	932	770	383	794	634	820	180	0
Industrial	2301	5287	11891	987	2971	6116	10460	8517	1885	8908	6233	4805	6448
Land(1)	523	778	4413	954	450	3880	1932	2136	863	1882	1959	865	2630
Land(2)	163	290	647	389	1116	1059	482	482	410	662	3156	82	1216
Land(3)	2202	428	5339	2052	1929	9224	7641	5830	5134	1667	5205	1014	5996
Land(4)	995	1744	5141	1014	885	3043	2863	2031	1487	1263	2060	782	2096
Livestock	106	256	120	89	31	4045	1745	106	528	2084	3809	43	1358
Aquaculture	0	12	467	19	234	0	118	43	815	3153	26126	0	3875

Domestic sources refer to (1) sewage treatment plants, (2) combined septic tanks, (3) individual septic tanks, (4) human waste treatment plants, (5) treated wastewater, (6) untreated wastewater. Land sources refer to (1) paddies, (2) fields/orchards, (3) mountains/forests, (4) the other lands.

Table S3 The reported T-P pollutant loads (kg/day) by prefecture used in the method described in Table 1.

Categories of sources	Kyo.	Osa.	Hyo.	Nar.	Wak.	Oka.	Hir.	Yam.	Tok.	Kag.	Ehi.	Fuk.	Oit.
Domestic(1)	617	1206	1148	214	42	180	544	355	32	54	181	196	152
Domestic(2)	8	25	97	22	53	45	49	27	39	13	24	20	33
Domestic(3)	0	0	1	1	1	4	0	0	4	0	1	0	0
Domestic(4)	3	0	1	0	1	3	1	0	0	0	0	0	1
Domestic(5)	0	126	0	97	0	0	16	20	0	8	0	0	109
Domestic(6)	19	0	54	0	116	139	112	56	116	92	120	26	0
Industrial	318	356	544	171	408	461	496	686	385	358	483	130	478
Land(1)	6	10	58	12	5	51	25	28	11	24	25	11	34
Land(2)	2	3	8	5	14	13	6	6	5	8	41	1	16
Land(3)	58	28	140	54	50	242	201	153	135	43	137	26	157
Land(4)	26	59	135	26	23	80	75	53	39	33	54	20	55
Livestock	6	90	7	6	3	150	289	16	71	144	661	4	330
Aquaculture	0	9	121	5	61	0	33	10	153	345	2173	0	588

Domestic sources refer to (1) sewage treatment plants, (2) combined septic tanks, (3) individual septic tanks, (4) human waste treatment plants, (5) treated wastewater, (6) untreated wastewater. Land sources refer to (1) paddies, (2) fields/orchards, (3) mountains/forests, (4) the other lands.

Table S4 The correlation coefficient between the rate of decrease in the amount of generated loads and the rate of depopulation by sea area from 2020 to 2050.

	**T-N**	**T-P**
Considering all the sea areas	-0.2247	-0.3131
Considering 11 sea areas excluding Osaka Bay	-0.2074	-0.3484
Considering 11 sea areas excluding Bungo Channel	0.6222	0.5450

Table S5 The runoff rate to the sea area in the report.

Prefecture	T-N(%)	T-P(%)
Kyoto	32.2	16.4
Osaka	23.7	16.5
Hyogo	20.1	15.8
Nara	25.1	15.9
Wakayama	21.5	14.9
Okayama	16.4	12.1
Hiroshima	23.1	16.1
Yamaguchi	20.4	14.7
Tokushima	23.1	17.3
Kagawa	21.9	15.0
Ehime	21.9	15.3
Fukuoka	16.1	11.2
Oita	20.9	15.1

The runoff rate used when converting the amount of pollutant load into the amount of load flowing into the sea is presented in Table S3. This value was taken from a report by the Ministry of the Environment[40].
